# A Rapid and Accurate Extraction Procedure for Analysing Free Amino Acids in Meat Samples by GC-MS

**DOI:** 10.1155/2015/209214

**Published:** 2015-03-19

**Authors:** Trinidad Pérez-Palacios, Miguel A. Barroso, Jorge Ruiz, Teresa Antequera

**Affiliations:** ^1^Department of Animal Production and Food Science, Faculty of Veterinary Sciences, University of Extremadura, Avenida de la Universidad s/n, 10003 Cáceres, Spain; ^2^Dairy, Meat and Plant Product Technology, Department of Food Science, University of Copenhagen, Rolighedsvej 30, 1958 Frederiksberg C, Denmark

## Abstract

This study evaluated the use of a mixer mill as the homogenization tool for the extraction of free amino acids in meat samples, with the main goal of analyzing a large number of samples in the shortest time and minimizing sample amount and solvent volume. Ground samples (0.2 g) were mixed with 1.5 mL HCl 0.1 M and homogenized in the mixer mill. The final biphasic system was separated by centrifugation. The supernatant was deproteinized, derivatized and analyzed by gas chromatography. This procedure showed a high extracting ability, especially in samples with high free amino acid content (recovery = 88.73–104.94%). It also showed a low limit of detection and quantification (3.8 · 10^−4^–6.6 · 10^−4^ *μ*g *μ*L^−1^ and 1.3 · 10^−3^–2.2 · 10^−2^ *μ*g *μ*L^−1^, resp.) for most amino acids, an adequate precision (2.15–20.15% for run-to-run), and a linear response for all amino acids (*R* ^2^ = 0.741–0.998) in the range of 1–100 *µ*g mL^−1^. Moreover, it takes less time and requires lower amount of sample and solvent than conventional techniques. Thus, this is a cost and time efficient tool for homogenizing in the extraction procedure of free amino acids from meat samples, being an adequate option for routine analysis.

## 1. Introduction

Meat and meat products are a good source of amino acids and their proteins are considered of high biological quality. After consumption of meat, free amino acids are rapidly absorbed, while proteins are easily hydrolysed into peptides and amino acids, which in turn are also absorbed. Apart from their nutritional importance, amino acids also influence meat palatability [1] and flavour [2], through the generation of volatile compounds by Maillard reactions and Strecker degradations [3–6]. During the processing of dry-cured meat products, such as dry-cured ham or loin, there happens an increase in free amino acid content as a consequence of proteolytic activity [4, 7]; indeed, the amount of most amino acids increases with processing time and with higher processing temperatures [3, 8, 9]. Glutamic acid and phenylalanine have been found to be the major amino acids in fresh meat, while in dry-cured products glutamic acid, arginine, and lysine have shown the highest levels [10].

Traditionally, the most common method to analyze free amino acids in food matrices has been reverse phase-high performance liquid chromatography (RP-HPLC) with a precolumn derivatization step [10]. Gas chromatography coupled with mass spectrometry (GC-MS) can be also used as an alternative method, especially when sample amounts are limited and high sensitivity is required [11]. In addition GC presents higher resolution and speed of analysis and lower instrumental cost than HPLC [12]. When GC was first used for the analysis of amino acids, its main drawback was the time consuming and tedious derivatization steps (esterification + acylation) required. Then, the simultaneous silylation of the amino and carboxyl groups in a single step, first using bis(trimethylsilyl)trifluoroacetamide (BSTFA) [13] and later with N-methyl-N-(tert-butyldimethylsilyl)trifluoroacetamide (MTBSTFA) [14], was developed. MTBSTFA allows the use of milder derivatization conditions [15]. Jiménez-Martín et al. [10] have demonstrated the suitability of using MTBSTFA for the determination of free amino acids in different animal source foods.

A previous free amino acid extraction is required before their derivatization and further analysis. Most extraction methods for amino acids in food products involve using perchloric acid or hydrochloric acid (HCl) diluted in water or in ethanol [9, 10, 12, 16–20]. Other solvents for the extraction of amino acids have also been described in the scientific literature, such as ethanol [21], and solvent mixtures, such as water/acetonitrile (50 : 50, v/v) [22] or 0.1% (v/v) formic acid in 20% (v/v) methanol [23]. After mixing the sample with the solvent, the homogenization step is essential for the amino acids to be extracted. For this purpose, different techniques have been used, that is, stirring [12], ultraturrax [16, 17], stomacher [10, 17], omni mixer [9], rotary mixer at 50°C [21], vortex [23], and a heating block at 40°C with stirring [18]. Then, centrifugation is usually carried out, followed by the collection of the supernatant [20] and its filtration through glass wool [9, 10], nylon membrane [23], or Whatman 42 paper [17]. Some authors clean up the supernatant through a cartridge [12, 21] and others do not specify the filtration procedure [9, 20].

The search for new and accurate methods for amino acid analysis in meat and meat products is challenging. The development of derivatization and chromatographic procedures has been thoroughly studied, while less attention has been paid to the extraction methods [24]. Recently, Jiménez-Martín et al. [10] described a GC-MS method for the determination of free amino acids in animal source food. In this methodology, the sample is homogenized with HCl 0.1 M by using a stomacher. Acetonitrile is used for deproteinizing and MTBSTFA for derivatizing. The application of this GC-MS method for the determination of amino acids in meat and meat products constitutes an important reduction in time and solvents in the separation and detection procedures in comparison to RP-HPLC with diode array detector method [3, 4, 8, 9]. However the extraction protocol is time consuming and requires a large amount of sample and solvent, which makes it frequently not suitable for routine analysis.

The present work is focused on the homogenization step for the amino acid extraction from meat samples, with the main goal of reducing sample amount, solvent volume, and extraction time. Recently, Segura and Lopez-Bote [25] developed a new procedure to extract intramuscular fat from pork based on homogenizing the samples using a mixer mill, which allowed minimizing the sample amount, the solvent use, and the analysis time, which are important advantages for routine analysis. The mixer mill is a compact versatile bench-top unit, which has been developed specially for homogenizing small amounts of sample quickly and efficiently by impact and friction. The grinding jars perform radial oscillations in a horizontal position. The inertia of the grinding balls causes them to impact with high energy on the sample material at the rounded ends of the grinding jars and pulverizes it. Also, the movement of the grinding jars combined with the movement of the balls results in the intensive mixing of the sample. The degree of mixing can be increased even further by using several smaller balls.

Thus, the objective of this study was to evaluate the use of the mixer mill as homogenization tool in the extraction of free amino acids from meat samples, in order to analyze a large number of samples in the shortest time, minimizing sample amount and solvent volume.

## 2. Material and Methods

### 2.1. Samples

This study was developed with two different meat samples, fresh pork loin and dry-cured ham. These samples were obtained from a local store. First, samples were ground using a commercial grinder. Subsequently, the moisture content of the products was determined according to the method of the Association of Official Analytical Chemists [26] (moisture reference 935.29). The rest of the ground samples were stored at −80°C until free amino acid analysis.

### 2.2. Reagents

Hydrochloric acid (HCl), 37% extra pure, was used for the amino acid extraction (Scharlau, Barcelona, Spain). Acetonitrile of HPLC-gradient grade (Panreac, Barcelona, Spain) and dichloromethane (Merck, Darmstadt, Germany) were used for the amino acid deproteinization and derivatization procedures. MTBSTFA (Sigma-Aldrich, Madrid, Spain) was the derivatization reagent. Standard amino acids (Sigma-Aldrich) purchased for preparing the standard solutions were alanine, glycine, valine, leucine, isoleucine, proline, methionine, serine, threonine, phenylalanine, aspartic acid, hydroxyproline, cysteine, glutamic acid, arginine, asparagine, lysine, glutamine, histidine, tyrosine, tryptophan, and cystine. DL-Norleucine (Sigma-Aldrich) was used as internal standard (IS).

### 2.3. Amino Acid Extraction Methods

Two methods for the amino acid extraction were compared. They mainly differ in the homogenization procedure, carried out with stomacher (*S*) or mixer mill (*M*). Both methods used HCl 0.1 M as solvent extraction and the sample : solvent ratio was 1 : 7.5. The free amino acid content of fresh loin (*n* = 6) and dry-cured ham (*n* = 6) was analysed by using the two extraction methods. Each sample was analysed in triplicate.

#### 2.3.1. Stomacher Method

Samples (2 g) were weighed, mixed with HCl 0.1 M (15 mL), and subsequently homogenized in a stomacher (Stomacher 400, Lab-Blender, Barcelona, Spain) for 4 min, as described by Jiménez-Martín et al. [10]. From the stomacher bag, 2 mL was transferred to a safe-lock micro test tube and centrifuged (10000 rpm) (Eppendorf Centrifuges, model 5810R) for 15 minutes at 4°C. The supernatant was stored at −80°C until analysis.

#### 2.3.2. Mixer Mill Method

Ground samples (0.2 g) were homogenized with HCl 0.1 M (1.5 mL) and three stainless steel balls (2 mm of diameter) in the mixer mill (MM400, Retsch technology, Haan, Germany) during 2 min and centrifuged (10000 rpm, 15 min, 4°C). After then, the supernatant was stored at −80°C until analysis.

### 2.4. Deproteinization and Derivatization

To deproteinize the sample, 250 *μ*L of acetonitrile was mixed with 100 *μ*L of the extract in safe-lock micro test tube and centrifuged at 10000 rpm for 3 min. 100 *μ*L of the supernatant was transferred to heat-resistant tubes and 100 *μ*L of IS solution (5 *μ*g mL^−1^) was added. Then, tubes were dried under nitrogen. The residual water was removed adding 50 *µ*L of dichloromethane to the dried samples and again evaporated under nitrogen. Finally, 50 *μ*L of MTBSTFA and 50 *μ*L of acetonitrile were added to the dried tubes, which were shaken and subsequently incubated at 100°C for 60 min to induce the derivatization reaction to occur. Then, tubes where stored at refrigeration and analyzed by GC-MS within the next 24 hours.

### 2.5. Instrumentation

The chromatographic analysis was carried out in a GC equipment 5890 series II (Hewlett-Packard, Barcelona, Spain) coupled to a mass selective detector (MSD) electron impact (EI), model 5973 (Agilent, Barcelona, Spain). A 1 *μ*L portion of the derivatized extract was injected in splitless mode onto the column. The column used was a 50 m × 0.32 mm i.d., 1.05 *μ*m, HP-5 (Hewlett-Packard), being a 5% phenyl-methyl polysiloxane bonded phase fused silica capillary column. Column head pressure was 12.8 psi, resulting in a flow of 1.2 mL/min at 280°C. The oven program was as follows: 170°C for 5 min, 4°C/min ramp to 200°C, held at 200°C for 3 min, 4°C/min ramp to 290°C, held at 290°C for 1 min, 20°C/min ramp to a final temperature of 325°C, and held for 15 min. The transfer line to the mass spectrometer program was as follows: 280°C for 35 min, 10°C/min ramp to 320°C. Total run time was 55.75 min. Free amino acids were identified both by their retention time and by comparison of their characteristic* m/z* ions with those published in the literature [9, 10]. The quantification was carried out in the selected ion monitoring (SIM) mode.  Table 1 shows retention time (Rt), ions selected in SIM mode, and the selected ion for quantification of each amino acid in this study. A calibration curve (quantification ion AA peak area/quantification ion IS peak area versus AA amount/IS amount) was constructed, obtaining *R* ^2^ values of 0.9999. The final results, expressed in microgram per 100 gram sample dry weight, take into account the moisture content and the exact weight of the sample.

### 2.6. Standard and Calibration Curves

A standard calibration solution containing 200 *µ*g mL^−1^ for each AA was prepared (0.5 g of each amino acid was dissolved in 250 mL of HCl 0.1 M). From this solution, seven decreasing dilutions were made (150, 100, 50, 25, 10, 5, and 1 *μ*g mL^−1^). A stock solution of IS at 5 *μ*g mL^−1^ was prepared in 0.1 M HCl.

### 2.7. Quality Control

Quality control of the GC-MS analysis was performed through the routine analysis of procedural blanks and quality control standards and samples to ensure the absence of contaminants and the possible carryover between samples and to assess the quality of the results. Limit of detection (LOD) and quantification (LOQ) based on a signal/noise ratio of 3 : 1 and 10 : 1, respectively, were determined using aqueous standard solutions (*n* = 5) with the following equations: LOD = 3SD/*b* and LOQ = 10SD/*b*, where, for each free amino acid, SD is the standard deviation of the average of the signal obtained for the calibration solution of lowest concentration (0.1 mg/100 mL) and *b* is the slope of the analytical curve calculated with the calibration solutions. For calculating the relative standard deviation (RSD) run-to-run, five replicate analyses of samples were done. In these determinations, ions were selected in SIM mode.

In order to study the recovery for each AA, loin and dry-cured ham samples were spiked with appropriate amounts of AA (7.5–40 *μ*g) each and were extracted using *S* and *M* methods. Moreover, the recovery was also calculated in unspiked samples, using the aqueous standard solutions.

### 2.8. Statistical Analysis

The effect of the extraction method on total chromatographic area as well as on the content of each detected amino acid was analysed by the Student's* t*-test for independent samples. Linear regression analysis was carried out in order to compare the response of the different homogenization tools. The SPSS package (v 18.0) was used.

## 3. Results and Discussion

### 3.1. Evaluation of the Mixer Mill as Homogenization Tool for Free Amino Acid Extraction

**Figure 1:**
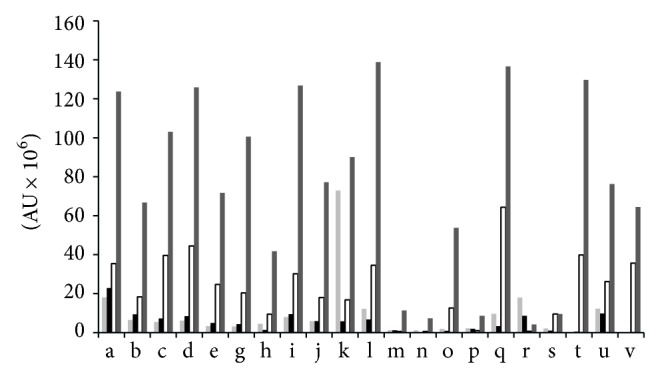
Area units (AU) of chromatographic area of each free amino acid detected in fresh loin and dry-cured ham samples by using stomacher (light grey and white, resp.) and mixer mill (black and dark grey, resp.) as homogenization tools. Alanine (a), glycine (b), valine (c), leucine (d), isoleucine (e), proline (g), methionine (h), serine (i), threonine (j), phenylalanine (k), aspartic acid (l), hydroxyproline (m), cystine (n), glutamic acid (o), asparagine (p), lysine (q), glutamine (r), arginine (s), histidine (t), tyrosine (u), and tryptophan (v).

The chromatographic areas of each free amino acid detected in fresh loin and dry-cured ham samples homogenized by using *S* and *M* are shown in Figure 1. Most AA showed no statistical differences in fresh loin between *S* and *M*, whereas in dry-cured hams chromatographic areas of free amino acids were significantly higher (*P* < 0.05) when using *M* than *S* for extraction.

**Figure 2:**
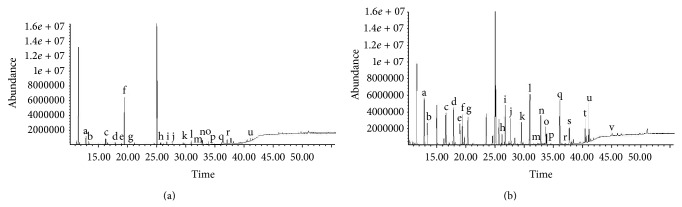
Chromatogram of free amino acids detected in fresh loin (a) and dry-cured ham (b) samples. Alanine (a), glycine (b), valine (c), leucine (d), isoleucine (e), norleucine (f, internal standard), proline (g), methionine (h), serine (i), threonine (j), phenylalanine (k), aspartic acid (l), hydroxyproline (m), cystine (n), glutamic acid (o), asparagine (p), lysine (q), glutamine (r), arginine (s), histidine (t), tyrosine (u), and tryptophan (v).

Figure 2 shows a GC-MS chromatogram of the free amino acids detected in fresh loin (Figure 2(a)) and dry-cured ham (Figure 2(b)) when using *M*. Twenty-one free amino acids were detected in dry-cured samples: alanine, glycine, valine, leucine, isoleucine, proline, methionine, serine, threonine, phenylalanine, aspartic acid, hydroxyproline, cysteine, glutamic acid, asparagine, lysine, glutamine, arginine, histidine, tyrosine, and tryptophan, while fresh loin samples presented 18 free amino acids, the same as dry-cured hams except for arginine, histidine, and tryptophan.

Results on free amino acid content in fresh and dry-cured hams using *S* and *M* homogenization tools are shown in Table 2. As expected, most amino acids showed a higher content in dry-cured ham than in fresh loin, which is in agreement with previous results [10]. This can be ascribed to the longer time during which the proteolytic activity takes place in the processing of the hams [4, 8, 9].

Content of most amino acids from fresh loin did not show statistical differences between *S* and *M*, and nor did the sum of total amino acids. However, hydroxyproline and glutamic acid were only detected when using *M*. In addition, the profile of free amino acid did not vary with the homogenization method. Major free amino acids in loins were glutamine (148.33 and 153.93 mg/100 g sample dry matter in *S* and *M*, resp.), cysteine (100.99 and 103.71 mg per 100 g sample dry matter in *S* and *M*, resp.), while leucine (12.22 and 11.77 mg per 100 g simple dry matter in *S* and *M*, resp.), isoleucine (11.30 and 10.75 mg per 100 g simple dry matter in *S* and *M*, resp.), hydroxyproline (nondetected and 10.24 mg per 100 g simple dry matter in *S* and *M*, resp.), and valine (11.20 and 8.80 mg per 100 g simple dry matter in *S* and *M*, resp.) showed the lowest content. The levels of the other amino acids detected in fresh loin were between 29 and 69 mg per 100 g sample dry matter. These results are in agreement with previous findings. Glutamine has been described as the major amino acid in fresh meat [27, 28]. In relation to the tryptophan, it has been detected in low concentration in fresh meat [29]. According to results found by Jiménez-Martín et al. [10], glutamic acid is the major amino acid in fresh pork, followed by glutamine, cysteine, and phenylalanine.

In dry-cured ham samples, most amino acids detected and the sum of total amino acids showed higher content when using *M* for extraction as compared to *S*. The *M* procedure could break more effectively the meat gel structure formed during the processing of the dry-cured hams than the *S* one. In fact, other authors [29, 30] observed a difficulty of protein extraction during the processing of Iberian hams, even using solutions with high ionic strength for their extraction. The observed suitability of the *M* in the extraction procedures for the analyses of these compounds could be related to the combined movement of the grinding jars with the balls, which results in an intensive mixing of the ham sample with the solvent.

The obtained results highlight the accuracy of the *M* homogenization tool, which is crucial in the case of samples containing high amino acid content, as dry-cured hams do. This is in concordance with results found by Segura and Lopez-Bote [25], who tested the mixer mill for the extraction of intramuscular fat. These authors noticed that the higher extracting ability of the mixer mill was more evident in samples with high levels of intramuscular fat rather than in low lipid content ones.

In spite of the influence of the homogenization tool on the free amino acid content in dry-cured hams, the overall profile of amino acids was similar with *M* and *S* extraction methods. Glutamic acid (352.89 and 520.02 mg per 100 g sample dry matter in *S* and *M*, resp.) and lysine (356.36 and 554.91 mg per 100 g sample dry matter in *S* and *M*, resp.) were the major amino acids in dry-cured ham samples, with hydroxyproline (14.45 and 20.48 mg per 100 g sample dry in *S* and *M*, matter, resp.) and asparagine (47.83 and 37.09 mg per 100 g sample dry in *S* and *M*, matter, resp.) being the minor ones. The content of the other amino acids in dry-cured ham was between 54 and 307 mg per 100 g sample dry matter. Previous research on dry-cured ham showed similar results [4, 8–10]. Nevertheless, considering results from different works, it can be noticed a high variability in the content of some amino acids from hams; that is, Jurado et al. [7] found higher content of glutamic acid (1269 mg per 100 g sample dry matter) than Martín et al. [4] (650 mg per 100 g sample dry matter), Pérez-Palacios et al. [9] (271 mg per 100 g sample dry matter), and Jiménez-Martín et al. [10] (271 mg per 100 g sample dry matter). These differences may be ascribed to the different processing of hams (salting time, temperature, and moisture conditions). Moreover, several factors may affect aminopeptidase activity during dry-cured ham processing, such as sodium chloride, which is a potent inhibitor for these enzymes [31]. In addition, the water loss and the subsequent reduction in water activity that takes place during dry-cured ham processing also influence the proteolytic activity [32]. Free amino acid accumulation has a feedback effect, reducing aminopeptidase activity [33]. Finally, the variability in the content of free amino acids among works can be also related to the differences in the extraction method. In fact, this work shows significant differences in the content of amino acids of the same samples analyzed under the same conditions, except for the procedure of the extraction method.

Correlation analysis between amino acid content obtained using the *S* and *M* extraction methods was carried out in order to compare the response of two methodologies.  Table 3 shows regression equations and coefficient of determination for each amino acid detected. It can be observed that the response is linear for all amino acids (*R* ^2^ = 0.741–0.998), suggesting that the validity of the *M* homogenization tool is similar to that of *S* one, which has been previously validated [10].

Amount of sample and volume of solvent used and time consumed are notable aspects to take into account when comparing methodologies. At this respect, time analysis, sample quantity, and solvent volume for the extraction of free amino acids in twenty samples by using *S* and *M* homogenization tools were estimated (Table 4). *M* takes less time and requires lower amount of sample and solvent than *S* (2 versus 80 min, 4 versus 40 g, and 30 versus 300 mL, resp.).

The observed ability of the *M* method in the homogenization process for extraction of free amino acids in meat samples, reducing notably sample and solvent amount as well as time consuming, makes it appropriate for routine analysis.

### 3.2. Quality Control

The performance of the GC-MS method was examined by determining quality parameters for each individual amino acid. Good linearity was obtained for the range 1–100 *μ*g mL^−1^ for the 22 standard amino acids. The correlation coefficients were >0.90, except for tryptophan (*R* ^2^ = 0.837). Most amino acids showed a poor linearity above 150 *μ*g mL^−1^; thus, curve point at this concentration or higher was avoided. A similar behaviour was reported previously [10]. LOD and LOQ of the analytical procedure ranged from 3.8·10^−4^–6.6·10^−4^ *μ*g *μ*L^−1^ to 1.3·10^−3^–2.2·10^−2^ *μ*g *μ*L^−1^, respectively, for alanine, glycine, valine, leucine, isoleucine, proline, methionine, serine, threonine, phenylalanine, aspartic acid, histidine, and tyrosine. For glutamine, asparagine, lysine, glutamic acid, tryptophan, and cysteine these values were around 0.02–0.2 *µ*g *µ*L^−1^ and 0.07–0.66 *µ*g *µ*L^−1^ for LOD and LOQ, respectively. These results are quite in concordance with previous studies [10, 24]. Hydroxyproline and cysteine had higher values for LOD (0.38 and 1.27 *µ*g *µ*L^−1^, resp.) and LOQ (0.98 and 2.98 *µ*g *µ*L^−1^, resp.). In fact, previous studies using RP-HPLC-DAD for analyzing amino acids from dry-cured hams did not allow the detection of hydroxyproline and cysteine [8]. Adequate precision was achieved with a RSD of 2.15–20.15% for run-to-run.

Table 5 shows the recovery of AA in aqueous standard solution and in spiked samples (loin and dry-cured ham) extracted by using both *S* and *M* extraction methods. In aqueous standard solution all AA showed high recoveries (94.49–105.75%), indicating the accuracy of the chromatographic procedure. In the samples, most AA showed higher recoveries when using the *M* method for the extraction in comparison to the *S* one, especially in dry-cured ham. This result points out the suitability of the mixer mill for the extraction of AA and it is in concordance with other studies in AA from meat samples [34].

## 4. Conclusions

The mixer mill is an appropriate tool for the homogenization step in the extraction procedure of free amino acids from meat samples, especially in samples with high free amino acids content. In addition, this technique notably reduces sample amount and solvent volume as well as analysis time. Thus, it could be an adequate option for routine analysis of free AA in meat and meat products.

## Figures and Tables

**Table 1:** Ions selected in SIM mode (quantification ions in bold) and retention time (Rt) for the analysis of free amino acids.

Aminoacid	Rt (min)	Ions (*m*/*z*)
Alanine	12.86	**158**, 260, 232
Glycine	13.37	**218**, 246
Valine	16.60	**186**, 288, 260
Leucine	17.89	**200**, 302, 274
Isoleucine	18.98	**200**, 302, 274
Norleucine (IS)	19.45	**200**, 147, 274
Proline	20.38	**184**, 286, 258
Methionine	26.20	**218**, 320, 292
Serine	26.76	**362**, 390
Threonine	27.69	**404**, 376, 303
Phenylalanine	29.56	**336**, 302, 234
Aspartic acid	30.98	**316**, 418, 390
Hydroxyproline	31.78	**388**, 416, 314
Cysteine	32.35	**406**, 378
Glutamic acid	33.73	**432**, 330, 272
Asparagine	34.54	**417**, 302
Lysine	36.14	**300**, 431, 329
Glutamine	37.14	**329**, 431, 357, 338
Arginine	38.48	**442**, 340
Histidine	40.49	**196**, 489, 440, 338
Tyrosine	41.09	**466**, 438, 364, 302
Tryptophan	45.10	**244**, 489, 302
Cystine	50.18	**639**, 589, 537, 348

**Table 2:** Amino acid content (mg per 100 g sample dry matter) extracted from fresh loin and dry-cured ham samples by using two extraction procedures, with stomacher (*S*) and mixer mill (*M*).

	Fresh loin			Dry-cured ham			*P* (fresh loin versus dry-cured ham)
	*S*	*M*	*P*	*S*	*M*	*P*	
Alanine	41.82 ± 15.38	52.43 ± 19.48	0.500	234.86 ± 31.25	307.04 ± 22.74	0.032	<0.001
Glycine	<LOQ.	<LOQ.	—	69.49 ± 11.51	113.69 ± 10.23	0.008	<0.001
Valine	11.20 ± 1.63	8.80 ± 3.04	0.295	177.39 ± 28.43	242.10 ± 5.44	0.018	<0.001
Leucine	12.22 ± 2.10	11.77 ± 2.06	0.806	181.12 ± 29.86	254.25 ± 4.47	0.014	<0.001
Isoleucine	11.30 ± 1.45	10.75 ± 1.81	0.705	130.22 ± 20.76	174.52 ± 2.58	0.021	<0.001
Proline	36.74 ± 1.12	29.64 ± 0.97	0.001	186.77 ± 24.13	244.28 ± 15.73	0.026	<0.001
Methionine	29.38 ± 2.94	30.26 ± 9.03	0.881	77.04 ± 11.38	99.87 ± 0.41	0.026	<0.001
Serine	30.40 ± 1.46	27.98 ± 1.41	0.108	156.65 ± 30.08	244.85 ± 21.07	0.014	<0.001
Threonine	32.84 ± 2.87	37.13 ± 10.87	0.545	293.38 ± 66.02	488.86 ± 31.09	0.010	<0.001
Phenylalanine	28.23 ± 0.96	27.48 ± 4.83	0.804	118.40 ± 20.92	172.00 ± 2.26	0.012	<0.001
Aspartic acid	43.67 ± 4.24	43.43 ± 6.56	0.960	201.95 ± 38.71	281.37 ± 11.33	0.027	<0.001
Hydroxyproline	n.d.	10.24 ± 2.34	0.002	17.45 ± 1.17	20.48 ± 2.01	0.087	<0.001
Cysteine	100.99 ± 22.64	103.71 ± 26.27	0.898	154.55 ± 29.13	206.79 ± 23.52	0.073	0.001
Glutamic acid	n.d.	42.28 ± 2.86	<0.001	352.89 ± 80.64	520.02 ± 11.65	0.024	<0.001
Asparagine	56.75 ± 1.06	44.28 ± 2.10	0.001	47.83 ± 2.29	37.09 ± 0.90	0.002	0.059
Lysine	69.53 ± 8.15	48.94 ± 3.35	0.016	356.36 ± 91.72	554.92 ± 109.00	0.073	<0.001
Glutamine	148.33 ± 21.24	153.93 ± 28.16	0.797	69.34 ± 1.12	54.27 ± 0.90	<0.001	<0.001
Arginine	n.d.	n.d.	—	130.22 ± 8.08	224.19 ± 4.81	<0.001	<0.001
Histidine	n.d.	n.d.	—	119.13 ± 5.96	145.60 ± 7.29	0.008	<0.001
Tyrosine	66.39 ± 12.21	46.71 ± 6.01	0.066	91.05 ± 12.70	119.52 ± 2.73	0.019	<0.001
Tryptophan	n.d.	n.d.	—	26.82 ± 7.49	207.58 ± 3.76	<0.001	—
Cystine	n.d.	n.d.	—	n.d.	n.d.	—	—

LOQ: limit of quantification.

n.d.: not detected.

**Table 3:** Regression equations and coefficient of determination (*R* ^2^) between the content of each amino acid extracted with stomacher (*S*) and mixer mill (*M*) as homogenization tools.

Aminoacid	Regression equation	*R* ^2^
Alanine	*y*_*M*_ = 1.2674*x*_*S*_ + 4.4005	0.946
Glycine	*y*_*M*_ = 1.5921*x*_*S*_ + 1.5241	0.971
Valine	*y*_*M*_ = 1.3580*x*_*S*_ − 2.6046	0.971
Leucine	*y*_*M*_ = 1.3769*x*_*S*_ − 0.0915	0.958
Isoleucine	*y*_*M*_ = 1.3245*x*_*S*_ − 1.0850	0.962
Proline	*y*_*M*_ = 1.3859*x*_*S*_ − 17.9915	0.965
Methionine	*y*_*M*_ = 1.3385*x*_*S*_ − 6.1658	0.888
Serine	*y*_*M*_ = 1.6283*x*_*S*_ − 15.8795	0.955
Threonine	*y*_*M*_ = 1.6032*x*_*S*_ − 1.4932	0.922
Phenylalanine	*y*_*M*_ = 1.4914*x*_*S*_ − 9.6092	0.926
Aspartic acid	*y*_*M*_ = 1.4050*x*_*S*_ − 10.1634	0.941
Hydroxyproline	*y*_*M*_ = 0.5893*x*_*S*_ + 10.2204	0.905
Cysteine	*y*_*M*_ = 1.4952*x*_*S*_ − 37.4279	0.741
Glutamic acid	*y*_*M*_ = 1.2701*x*_*S*_ + 57.0415	0.941
Asparagine	*y*_*M*_ = 0.8251*x*_*S*_ − 2.7964	0.841
Lysine	*y*_*M*_ = 1.5545*x*_*S*_ − 29.1033	0.832
Glutamine	*y*_*M*_ = 1.2664*x*_*S*_ − 33.7431	0.998
Arginine	*y*_*M*_ = 1.7126*x*_*S*_ + 0.5797	0.994
Histidine	*y*_*M*_ = 1.2203*x*_*S*_ + 0.1130	0.997
Tyrosine	*y*_*M*_ = 1.9083*x*_*S*_ − 72.6416	0.851
Tryptophan	*y*_*M*_ = 7.0101*x*_*S*_ + 9.7883	0.905

**Table 4:** Estimation of analysis time, amount of sample, and solvent volume for the extraction of free amino acids in twenty samples by using stomacher (*S*) and mixer mill (*M*).

	*S*	*M*
Time (min)	80	2
Sample (g)	40	4
Solvent volume (mL)	300	30

**Table 5:** Recoveries (%) in aqueous standard solution (ASS) and in spiked samples extracted by using stomacher (*S*) and mixer mil (*M*).

	ASS	Fresh loin		Dry-cured ham	
		*S*	*M*	*S*	*M*
Alanine	94.49	65.45	82.04	69.13	104.94
Glycine	99.17	69.95	98.94	85.96	90.99
Valine	96.96	91.10	71.62	71.83	104.71
Leucine	96.62	83.78	80.73	69.82	98.01
Isoleucine	97.72	87.70	83.46	71.13	98.35
Proline	105.75	90.45	78.62	72.20	102.96
Methionine	99.97	72.24	74.38	74.95	99.65
Serine	98.33	96.48	88.81	61.47	97.58
Threonine	95.99	66.45	75.13	54.97	94.74
Phenylalanine	99.90	85.49	83.21	68.14	98.79
Aspartic acid	102.18	85.70	101.23	73.05	95.66
Hydroxyproline	97.90	54.63	82.80	40.41	88.84
Cysteine	102.52	80.12	82.28	69.12	100.45
Glutamic acid	102.27	77.27	103.47	82.24	98.75
Asparagine	105.38	91.76	77.05	91.16	93.79
Lysine	104.34	91.53	64.44	57.43	92.89
Glutamine	100.78	79.56	82.56	90.66	85.62
Arginine	102.76	92.47	78.43	54.80	97.67
Histidine	104.79	90.27	74.04	85.52	98.60
Tyrosine	102.02	84.13	89.19	81.99	88.73
Tryptophan	95.06	93.14	87.74	79.39	98.14
Cystine	106.87	88.21	99.31	84.29	92.69
